# Crystal engineering with copper and melamine†

**DOI:** 10.1039/d1ra02903g

**Published:** 2021-07-07

**Authors:** Ignacio Bernabé Vírseda, Shiraz Ahmed Siddiqui, Alexander Prado-Roller, Michael Eisterer, Hidetsugu Shiozawa

**Affiliations:** J. Heyrovsky Institute of Physical Chemistry, Czech Academy of Sciences Dolejskova 3 182 23 Prague 8 Czech Republic hide.shiozawa@jh-inst.cas.cz +420-26605-3755; Faculty of Physics, University of Vienna Boltzmanngasse 5 1090 Vienna Austria hidetsugu.shiozawa@univie.ac.at +43-1-4277-9726 +43-1-4277-72601; Institute of Inorganic Chemistry, Faculty of Chemistry, University of Vienna Währinger Straße 42 Austria; Atominstitut, TU Wien Stadionallee 2 1020 Vienna Austria

## Abstract

Coordination complexes and polymers are central in inorganic and materials chemistry as a variety of metal centers and coordination geometries lead to a diverse range of interesting properties. Here, size and structure control of gem-like quality monocrystals is demonstrated at room temperature. Using the same set of precursors, the copper-to-melamine molar ratio is adjusted to synthesize either a novel coordination complex of dinuclear copper and melamine (Cu2M1), or a barely-studied coordination polymer of zigzag copper–chlorine chains (Cu4M1). Crystals of the former are dark green and square with a size up to 350 μm across. The latter is light green, octagonal, and as large as 5 mm across. The magnetic properties of both crystals reflect the low-dimensional arrangements of copper. The magnetic susceptibility of Cu2M1 is modelled with a spin-1/2 dimer, and that of Cu4M1 with a spin-1/2 one-dimensional Ising chain. Controlled synthesis of such quality magnetic crystals is a prerequisite for various magnetic and magneto-optical applications.

## Abstract 2

Size and structure control of gem-like quality monocrystals – copper–melamine coordination complex, and copper–chlorine coordination polymer – is demonstrated at room temperature.

## Graphical abstract

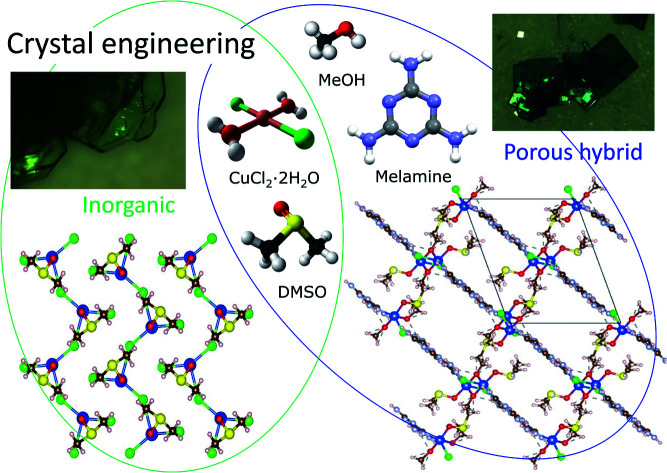

Advanced crystal engineering continues to draw the attention of the scientific community. This discipline is leading to new crystalline materials, and is focusing on finding strategies and logical ways to control their properties. Coordination chemistry plays an important role in crystal engineering as it allows the creation of various coordination compounds, *e.g.* metal complexes, coordination polymers or metal–organic frameworks, by designing the coordination between ligands and metal ions.^1–3^ While the d orbitals of the metal ions promote directional bonding, there has been widespread use of polyamines, carboxylates, pyridyl and cyano groups as ligands.^4^

Coordination compounds are considered useful in a great deal of applications, such as energy transfer, gas storage and separation, heterogeneous catalysis, proton conduction, biomedical applications and chemical sensing.^5,6^ Molecular magnets based on coordination compounds play an essential role in information storage in quantum computing.^7^ Single crystals based on Mn and Fe can serve as information storage elements in a dynamic random-access memory device in which decoding and reading the information could be realized by fast electron spin resonance pulses.^8^

In the present paper, it is demonstrated how molar ratios can affect reactions among precursor solutes and solvents. As an example, copper chloride and melamine are dissolved in a 1 : 1 mixture of methanol and dimethyl sulfoxide (DMSO) at room temperature. The structure of melamine is shown in Fig. 1b. Melamine-based coordination polymers reported previously include a fluorescent coordination polymer based on Cu(i) and melamine, which is highly stable and suitable for detection of nitro aromatic compounds in aqueous media.^9^ Also, a cationic coordination polymer based on Ag(i) and melamine was used for selective anion exchange.^10^

**Fig. 1:**
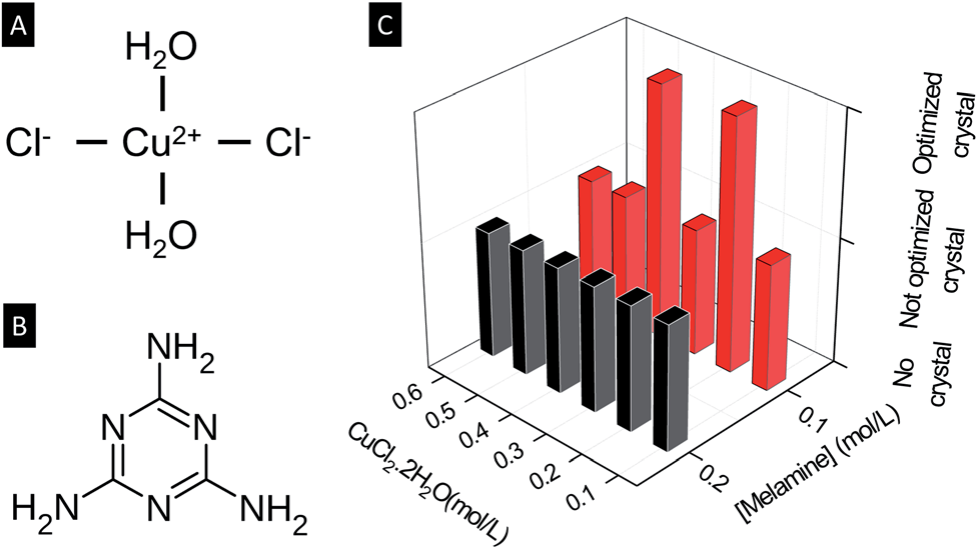
Schematic diagram of (A) CuCl_2_·2H_2_O and (B) melamine. (C) Crystal growth at room temperature with various concentrations of CuCl_2_·2H_2_O and melamine.

It is found that two kinds of large crystals grow at optimized concentrations and molar ratio between copper chloride and melamine. At an optimal concentration of melamine of 0.1 mol L^−1^, a copper to melamine ratio in the range 1 : 1 to 2 : 1, leads to the formation of a new copper complex composed of all available elements and molecules, *i.e.* copper, chlorine, melamine, methanol and DMSO. With a copper to melamine ratio in the range 3 : 1 to 4 : 1, both melamine and methanol are passivated, leading to the formation of a coordination polymer with zigzag copper–chlorine chains with each copper coordinated by three chlorines and two DMSO. Both of them are large single crystals with low-dimensional spin structures and are candidate materials for magnetic and sensing applications.

All synthesis was carried out at room temperature (around 25 °C) by mixing a methanol solution of CuCl_2_·2H_2_O and a DMSO solution of melamine at different concentrations and molar ratio. The concentrations of CuCl_2_·2H_2_O tested are 0.8, 0.6, 0.5, 0.4, 0.3, 0.2, 0.1, 0.05 and 0.025 mol L^−1^, and the concentrations of melamine are 0.05, 0.1 and 0.2 mol L^−1^. See ESI 1† for more details on the synthesis procedures and optimization. In each case, 2 mL of a methanol solution of CuCl_2_ was placed in a 5 mL glass vial, then 2 mL of a DMSO solution of melamine was added to the CuCl_2_ solution. The results are summarized qualitatively in Fig. 1 where ‘no crystal’ refers to the condition in which no solids are formed, while ‘not optimized crystal’ refers to the condition in which crystals grow but their quantity and/or size are not as large as the ‘optimized crystal’.

The two optimal conditions are (*ρ*_CuCl_2__, *ρ*_Melamine_) = (0.2 mol L^−1^, 0.1 mol L^−1^) for crystals named Cu2M1, and (0.4 mol L^−1^, 0.1 mol L^−1^) for crystals named Cu4M1, where *ρ*_CuCl_2__ is the concentration of CuCl_2_·2H_2_O in methanol and *ρ*_Melamine_ (mol L^−1^) is the concentration of melamine in DMSO. Crystals are formed only within the concentration window 0.05 mol L^−1^ ≤ *ρ*_CuCl_2__ ≤ 0.6 mol L^−1^ and 0.1 mol L^−1^ ≤ *ρ*_Melamine_ ≤ 0.2 mol L^−1^. The fact that no crystal growth can be achieved at high concentrations of both cations, Cu(ii) and anions Cl^−^, can be attributed to the high ionic strength that reduces the mobility of the ions in the solution,^11^ hindering the metal to ligand coordination. On the contrary, when the concentration is low, the mobility is high, but the nucleation of the crystal does not occur since the critical nucleation concentration is surpassed, and the collisions between both ligand and metal are less probable.^12^

Fig. 2 shows the micrographs of crystals Cu2M1 and Cu4M1, formed at the optimal conditions. For Cu2M1, dark green rectangular crystals with truncated edges are formed in 5 hours with sizes as large as 350 μm in length (see panels A, B and C). As for Cu4M1, light green octagonal crystals as large as 5 mm across are formed in 48 hours (panels D, E and F). This demonstrates that only doubling the concentration of CuCl_2_·2H_2_O can drastically alter the morphology of the formed crystal.

**Fig. 2:**
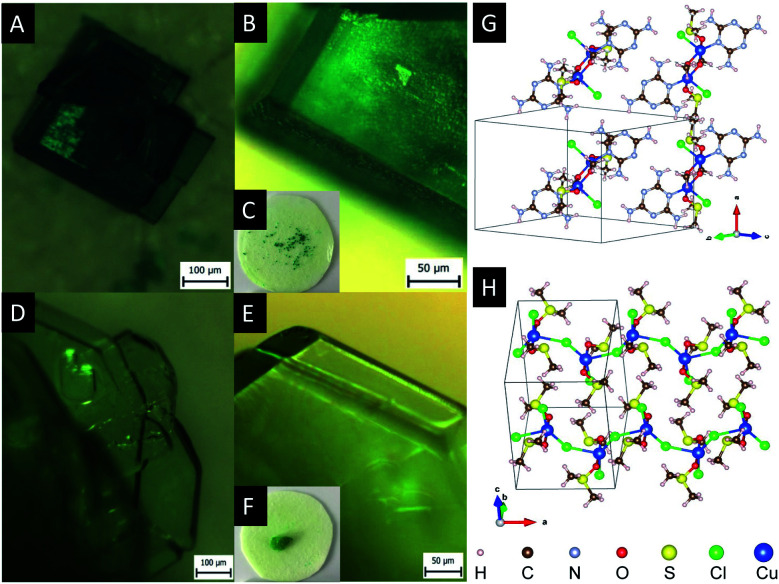
(A–C) Optical micrographs of Cu2M1: (*ρ*_CuCl_2__, *ρ*_Melamine_) = (0.2 mol L^−1^, 0.1 mol L^−1^). (D–F) Optical micrographs of Cu4M1 (*ρ*_CuCl_2__, *ρ*_Melamine_) = (0.4 mol L^−1^, 0.1 mol L^−1^). Panels C and F show crystals on the membrane with a diameter of about 2.5 cm. (G) The structure of Cu2M1 illustrating a layer of hydrogen-bonded melamine molecules in the (0 1 1) plane. (H) The structure of Cu4M1 illustrating zigzag Cu–Cl chains.

X-ray diffraction analysis of a Cu4M1 single crystal reveals the structure of the coordination complex (formula Cu((CH_3_)_2_SO)_2_Cl_2_)*, that was previously reported (CCDC deposition number 1142844),^13^ but not much was reported regarding its properties.^14,15^ The determined structure of Cu4M1, illustrated in Fig. 2H, consists of copper, chlorine and DMSO and is an orthorhombic system. As for its structure, it consists of serpent-like Cu–Cl chains. Each copper has a trigonal ligand geometry with a crystallographic point group of D_3h_ symmetry:^16^ bonded to two dimethyl sulfoxide molecules through a Cu–O bond with a length of 1.95 Å, forming a O–Cu–O angle of 173.67°, and to three chlorine atoms in the *a*–*c* plane. One of the chlorines is out of the zigzag chain and the other two are in the chain. Cl–Cu–Cl angles are 146.46°, 112.22° and 101.32°. The length of the three Cu–Cl bonds (2.75 Å) are longer than the covalent Cu–Cl bond length (2.3 Å) in CuCl_2_, indicating the weak covalent bonding.^13^Fig. 3 shows the inter- and intramolecular bonds. The bond lengths [Å] and the bond angles [°] are also given. It highlights that the zigzag chain results in a (red marked) “cap”. In the area marked in red, two other intramolecular bonds have also been detected (shaded green). The cap encloses a neighbouring strain and it is characterised by several intermolecular interactions (shaded yellow).

**Fig. 3:**
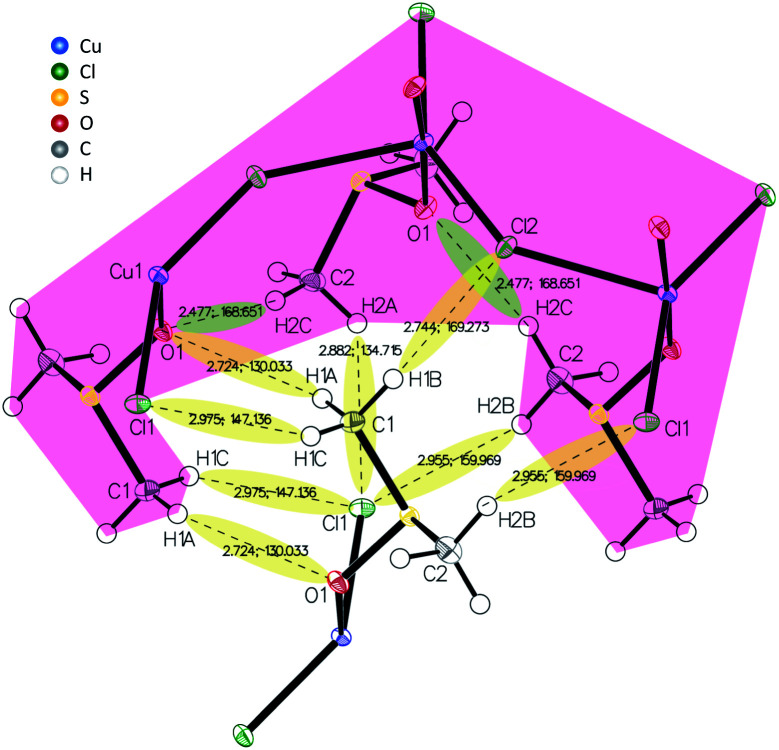
Inter- and intramolecular bonds visualized in the crystal structure of Cu4M1 drawn with 50% displacement ellipsoid.

X-ray diffraction analysis of a single crystal of Cu2M1 reveals that it is a coordination complex consisting of copper, chlorine, melamine, DMSO and methanol, as shown in Fig. 2G. The empirical formula of the coordination complex is (Cu(C_3_H_6_N_6_)(OCH_3_)((CH_3_)_2_SO)Cl)_2_. See ESI 2† for more details on the structural analysis. To the best of our knowledge, this crystalline structure has not been previously reported, only some similar examples as reported by Chen *et al.*, (2006) (CCDC deposition numbers 280091 and 280092),^17^ Goodgame *et al.*, (1999) (CCDC deposition number 134810),^18^ and Wiles *et al.*, (2006).^19^ In our Cu2M1 complex, two copper atoms are bridged by the oxygen atoms of two methoxides, forming an angle O–Cu–O of 77.88°. Each copper has a distorted square pyramidal ligand geometry with a crystallographic point group of C_4v_ symmetry.^16^ At the top of the square base pyramids is a chlorine Cu–Cl bond (2.63 Å). The basal plane of the pyramids consists of two Cu–O bonds (1.93 Å) where each copper coordinates with the oxygen of methoxide, another Cu–O bond (1.95 Å) which links copper with a molecule of DMSO by its oxygen, and a Cu–N bond (1.98 Å) that coordinates copper with a nitrogen of the pyridine ring of melamine. The two melamines are in the same plane which is outside of the basal plane of the two pyramids. Melamines of the adjacent Cu2M1 molecules are hydrogen bonded to one another, constituting a global two-dimensional layer of melamine. The packing view along (1 1 1) in Fig. 4 shows that every dimer (red shaded area) is surrounded by six neighbouring dimers in the plane. The shaded areas within the plane show the seven intermolecular interactions in yellow, and the two intramolecular interactions in green.

**Fig. 4:**
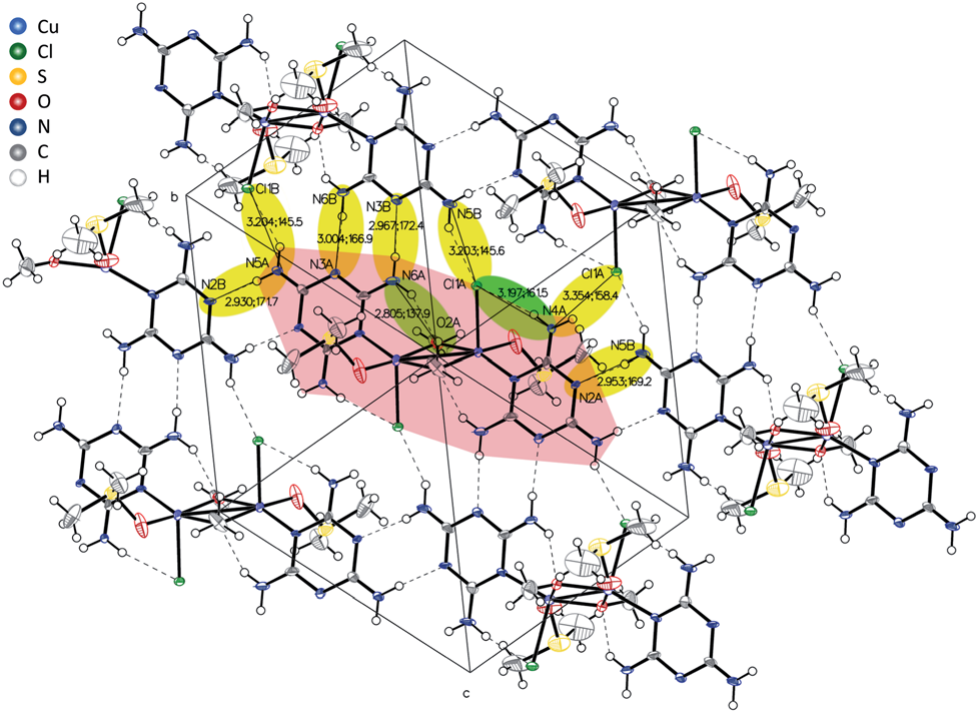
Inter- and intramolecular bonds visualized in the crystal structure of Cu2M1 drawn with 50% displacement ellipsoid.

Packing of melamine layers along (1 0 0) leads to two types of one-dimensional void accessible by the solvents (DMSO and MeOH). Fig. 5 compares the structure model for Cu2M1 viewed along the “*a*” axis without co-crystallised solvents in panel (a), with the methanol-filled model pictured in panel (b). The green-shaded void is along the visible radius in panel (a). This sterically influenced void allows the two different types of solvents (DMSO and MeOH) used during the synthesis to co-crystallise in a disordered way. The second type of void (yellow-shaded) is intruded by the coordinated DMSO and limits the available space in which only MeOH can be modelled. See ESI 2 for more details.†

**Fig. 5:**
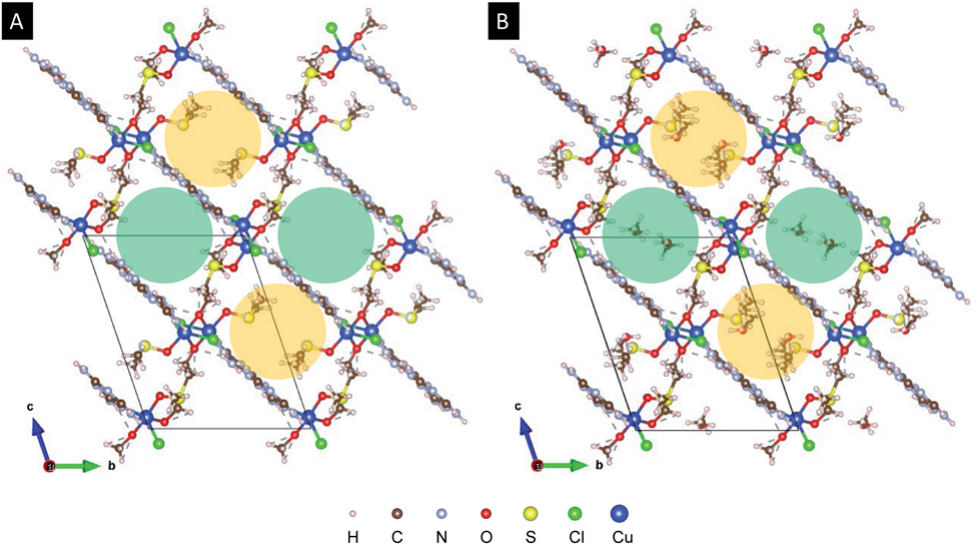
(A) The structure of Cu2M1 viewed along the *a* axis without filling. (B) The structure of Cu2M1 viewed along the *a* axis with methanol filling. The green and yellow-shaded circles represent the two types of one-dimensional void.

The crystal structures of Cu2M1 and Cu4M1 accommodate low-dimensional coordinations of copper, namely, the dinuclear copper molecular unit in Cu2M1 and the zigzag copper–chlorine chain in Cu4M1. The low-dimensional nature of the exchange coupling among copper spins are of particular interest for their potential magnetic applications.

The temperature dependence of the magnetic moment per copper in units of *μ*_B_ for a dinuclear Cu–melamine complex Cu2M1 sample (33.7 mg of Cu2M1 crystals encapsulated in a gelatin capsule) in an applied magnetic field of 1 T is shown in Fig. 6A. As planar crystals are stacked horizontally, the magnetic field is applied normal to the crystal plane for the majority of crystals. The net magnetism of the dinuclear Cu–melamine complex is much smaller than the dashed curve showing Curie’s Law for a paramagnetic 1/2 spin, with an effective magnetic moment of 
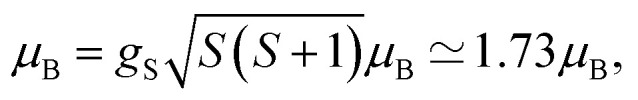
 indicating the presence of strong antiferromagnetic coupling. The magnetic susceptibility as a function of temperature can be analysed using the Bleaney–Bowers equation for an exchange-coupled pair of *S* = 1/2 spins^20,21^1
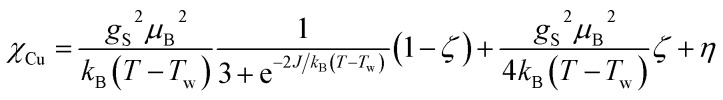
where, the first, second and third terms are the Bleaney–Bowers equation, Curie–Weiss law taking into account paramagnetic impurities, and temperature-independent constant *η*. Here, *g*_S_ ≃ 2 is the electron spin *g*-factor, *k*_B_ the Boltzmann constant, *J* the exchange energy between the spins, and *ζ* corresponds to a concentration of 1/2 paramagnetic impurities, *e.g.* isolated Cu(ii) on defects and surfaces. Weiss temperature *T*_w_, takes the molecular field due to intermolecular exchange into account. The least-squares fit is satisfactory with *J* = −0.0542 ± 0.0047 eV (≃−437 cm^−1^), *T*_w_ = −1.98 ± 0.05 [K], *ζ* = 0.158 ± 0.002 and *η* = 8.237 × 10^−4^ ± 2.11 × 10^−5^ [*μ*_B_/Cu]. In Fig. 6A, the Bleaney–Bowers fit magnified 1000 times is plotted. Because of the relatively high exchange constant, the Bleaney–Bowers component is small compared with the Curie–Weiss term in the measured temperature range 2–300 K, and reaches only about a half of the Curie’s component at room temperature. A negative Weiss temperature of *T*_w_ = −1.98 ± 0.05 [K] can be attributed to intermolecular anti-ferromagnetic coupling.

**Fig. 6:**
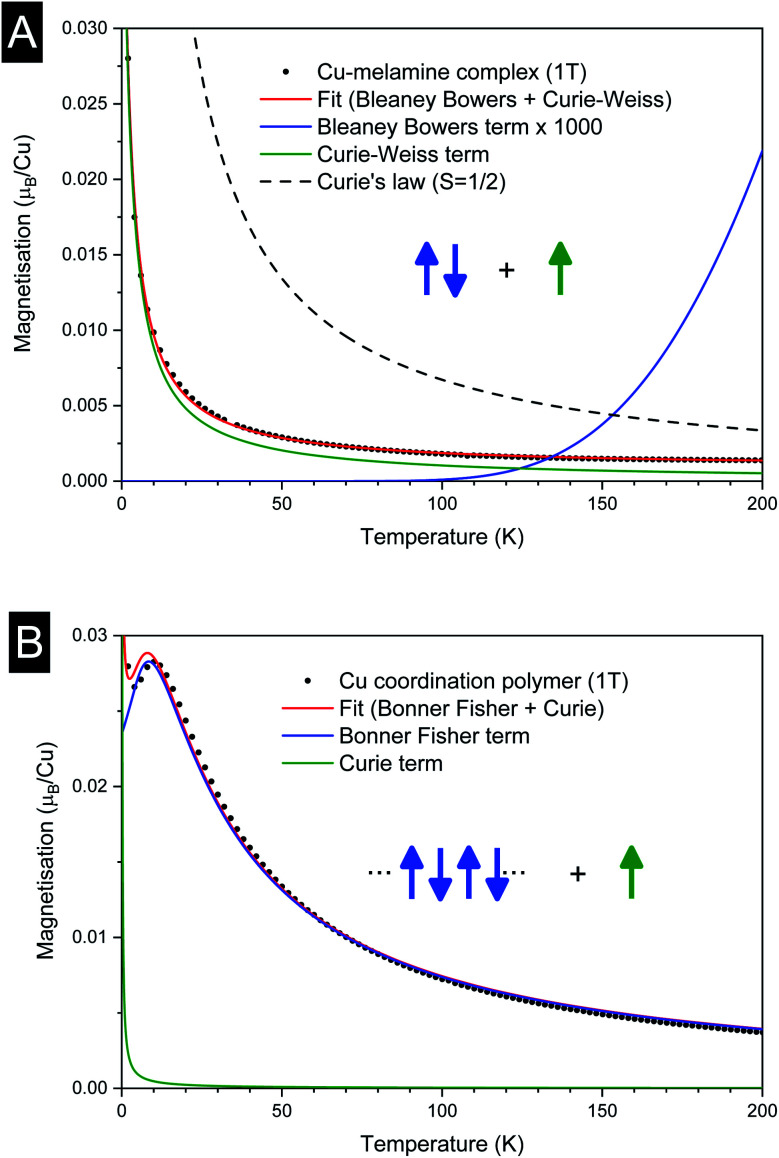
Magnetisation *versus* temperature for (A) dinuclear Cu–melamine complex Cu2M1, and (B) 1D-Cu coordination polymer Cu4M1.

As shown in Fig. 6B, the magnetization of a planar single crystal of copper coordination polymer Cu4M1 (24.0 mg) in the magnetic field applied normal to the crystal plane, exhibits a maximum of 0.0285 *μ*_B_ at 10 K. Provided that the exchange coupling is anisotropic and strong along the zigzag copper–chlorine–copper chain, the temperature dependence of the magnetic susceptibility can be evaluated based on the exchange Hamiltonian *Ĥ* = − *∑*_ij_*J*_ij_*Ŝ*_i_*Ŝ*_j_ taking exchange coupling between any two adjacent spins in a one-dimensional spin chain into account.^22–26^ The Bonner–Fisher equation derived from the above Hamiltonian for a *S* = 1/2 Heisenberg chain^27,28^ together with a paramagnetic term, is as follows in eqn (2),2
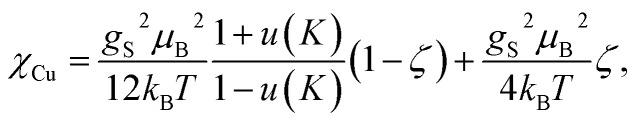
where *u*(*K*) = coth(*K*) − (1/*K*) and *K* = *J*/2*k*_B_*T*, and *ζ* corresponds to a concentration of 1/2 paramagnetic impurities or the inverse-temperature term that arises from staggered spins.^29^ The least-squares fit reproduces the experimental temperature dependence very well as shown in Fig. 6b, giving rise to *g* = 2.331 ± 0.005, *J* = −3.05 ± 0.02 meV (24.6 cm^−1^), *ζ* = 0.056 ± 0.0011. *g* is larger than 2 for pure spin states, but this value depends largely on the normalization of the data that may contain errors.

The structure analysis reveals that with a Cu–melamine ratio of 4 : 1 (0.4 mol L^−1^ of CuCl_2_·2H_2_O in methanol and 0.1 mol L^−1^ of melamine in DMSO) copper coordination polymer Cu4M1 is formed despite the presence of melamine. During their synthesis at room temperature, the pH of the media evolves differently in the 2 : 1 and 4 : 1 mixed solutions.^30^ Although evaluating pH values of organic and aprotic solvents is a complicated task, their relative changes upon chemical reactions are worth noting. Since there is no OH^−^ in the precursor solvents, the pH is only related to the presence of H^+^. The pH value of 0.2 mol L^−1^ of CuCl_2_·2H_2_O in methanol, 0.4 mol L^−1^ of CuCl_2_·2H_2_O in methanol and 0.1 mol L^−1^ of melamine before mixing are 1.03 ± 0.03, 0.62 ± 0.04 and 9.64 ± 0.02, respectively. The pH of the 2 : 1 and 4 : 1 solutions just after mixing are 5.96 ± 0.03 and 5.56 ± 0.02, respectively. After the formation of crystals of the dinuclear copper–melamine complex Cu2M1, the pH of the 2 : 1 solution remains unchanged within the confidence interval (5.94 ± 0.03). (This statistical parameter is determined by a “*t* of Student” distribution with 95% confidence interval, for which it is considered five simultaneous measurements.) On the contrary, the pH of the 4 : 1 solution is increased to 6.14 ± 0.03 after the formation of crystals of copper coordination polymer Cu4M1. This increase can be attributed to a reduction of H^+^ as a result of protonation of melamine which is initially deprotonated in pure DMSO. This leaves passivated neutral melamine which does not get coordinated with Cu(ii) ions. Likewise, the pH barely changes in the 2 : 1 solution because melamine ions react with Cu(ii) before being protonated. Hence, the proton concentration needs to be optimised for the formation of copper coordination polymer Cu4M1.

In order to justify the above-mentioned scenario, the synthesis of copper coordination polymer Cu4M1 has been attempted by mixing a methanol solution of CuCl_2_·2H_2_O with aprotic DMSO whose pH value is adjusted by adding anhydrous acetic acid. It is found that crystals of the same copper coordination polymer are formed in the solution without melamine when the pH is adjusted to 9.64 ± 0.02, while no crystals are formed without acetic acid. Thus, the concentration of protons plays an important role in the coordination of DMSO and chlorine with copper ions in the presence of methanol.

In summary, a novel copper–melamine complex and a copper coordination polymer have been synthesized selectively by adjusting the concentrations of copper(ii) chloride dihydrate and melamine in a mixed solution of methanol and DMSO at room temperature. Crystals of copper–melamine complex Cu2M1 formed with a Cu–melamine ratio of 2 : 1 are green and square shaped with sizes as large as 350 μm across. The hydrogen-bonded two-dimensional lattice is composed of planes of melamine and one-dimensional pores along the *a*-axis that accommodate solvent molecules. Crystals of copper coordination polymer Cu4M1 formed with a Cu–melamine ratio of 4 : 1 are light green gem-like octagonals and can grow as large as 5 mm across. The lattice is composed of Cu–Cl zigzag chains and has no porosity. Both Cu2M1 and Cu4M1 exhibit low-dimensional magnetic properties. The magnetic susceptibility of Cu2M1 can be modelled well based on the Hamiltonian for paired spins of 1/2, and that of Cu4M1 based on a spin-1/2 anti-ferromagnetic Ising chain. The well-controlled synthesis of the high quality and large monocrystals demonstrated in the present study will pave the way for future research on spintronic applications of inorganic and organic–inorganic hybrid materials.

## Conflicts of interest

There are no conflicts to declare.

## Supplementary Material

RA-011-D1RA02903G-s001

RA-011-D1RA02903G-s002

RA-011-D1RA02903G-s003
