# Supplementary material for: Crystal engineering with copper and melamine
Source: RSC Adv. 2021 Jul 7;11(39):23943–7. doi: 10.1039/d1ra02903g (PMC8262272; doi:10.1039/d1ra02903g)
Supplement: RA-011-D1RA02903G-s001 [file RA-011-D1RA02903G-s001.pdf]

Electronic Supplementary Material (ESI) for RSC Advances.
This journal is © The Royal Society of Chemistry 2021

Electronic Supplementary Information 1

Crystal engineering with copper and melamine

Ignacio Bernabé Vírseda,[a] Shiraz Ahmed Siddiqui, [b] Alexander Prado-Roller,[c] Michael Eisterer, [d] Hidetsugu Shiozawa*[a,b]

[a] *J. Heyrovsky Institute of Physical Chemistry, Czech Academy of Sciences, Dolejskova 3, 182 23 Prague 8, Czech Republic. Tel: +420-26605-3755; E-mail: hide.shiozawa@jh-inst.cas.cz*

[b] *Faculty of Physics, University of Vienna, Boltzmanngasse 5, 1090 Vienna, Austria. Fax: +43-1-4277-9726; Tel: +43-1-4277-72601; E-mail: hidetsugu.shiozawa@univie.ac.at*

[c] *Institute of Inorganic Chemistry, Faculty of Chemistry, University of Vienna, Währinger Straße 42, Austria*

[d] *Atominstitut, TU Wien, Stadionallee 2, 1020 Vienna, Austria*

Experimental

Chemicals

The ligand 1,3,5-Triazine-2,4,6-triamine (Melamine, C3H6N6, 99%) and the metal salt, copper chloride dihydrate ($CuCl_2 \cdot 2H_2O$, ≥ 99.0 %) were purchased from Sigma Aldrich.

As for the solvents employed, dimethyl sulfoxide (DMSO, $(CH_3)_2SO$, ≥ 99.7 %), N, N-Dimethylformamide anhydrous (DMF, $(CH_3)_2$-N-CHO, 99.8 %) and methanol (CH3OH, ≥ 99.9 %) were also purchased from Sigma Aldrich. Acetic acid (CH_3COOH, 99.0 %) from Penta and ammonia solution (NH_3, 4 mol/L, in methanol) from Sigma Aldrich were used. Deionized water (18.2 $M\Omega \cdot cm$) was used for all synthesis.

Optical microscope

Optical micrographs of crystals were taken with an optical microscope (BX41M-LED, Olympus, Japan) equipped with a Infinity 1 camera, Lumenera (Canada), and QuickPHOTO CAMERA 3.1 software.

Raman spectroscopy

Raman spectroscopy was performed using a Raman spectrometer (Model LabRAM HR, HORIBA, Japan with a He-Ne integrated laser (wavelength 633 nm).

Single crystal X-Ray diffraction

The X-ray intensity data were measured on Bruker D8 Venture diffractometer equipped with multilayer monochromator, Mo K/α INCOATEC micro focus sealed tube and Oxford cooling system.

Magnetization measurements

The magnetisation data were recorded at temperatures between 2K and 300K at applied magnetic fields up to 7T using a Quantum design MPMSXL magnetometer. 33.7 mg of

Cu2M1 crystals encapsulated in a gelatin capsule were measured. We assume that most of planar crystals lying horizontally were stuck on one another and hence the magnetic field was applied normal to the crystal plane. 24.0 mg of a planar single crystal of Cu4M1 was measured with the magnetic field applied normal to the crystal plane.

Synthesis

In this section, complementary information related to the optimization of the synthesis is represented. Three solutions of 0.05, 0.1 and 0.2 mol/L of melamine in DMSO are prepared. Solubility tests show DMSO, DMF, methanol and water can dissolve melamine, but only DMSO cannot fully dissolve if the concentration is above 0.2 mol/L at room temperature. As for the $CuCl_2 \cdot 2H_2O$ solutions, common solvents were tested: water, methanol, DMF and DMSO. Five mother solutions of 0.8, 0.6, 0.4, 0.3, 0.2, 0.1, 0.05 and 0.025 mol/L were prepared for each solvent system. In each case, 2 mL of a $CuCl_2$ solution was placed in a 5 mL glass vial, then 2 mL of a melamine solution was added to the $CuCl_2$ solution. The mixture was homogenized using a Transsonic T 470/H ultrasonic bath. After that, the vial was tightly closed and put aside at room temperature in a fume hood gas extractor for 3 days. The same procedure was followed for the syntheses at 50 °C in an oven, Ecocell model of BMT Medical Technology (Czech Republic). The main objective of this synthesis at a higher temperature is to determine the influence of temperature on the crystallization kinetics.

Figure S1-1 displays the results of crystal synthesis at room temperature with different solvents and $CuCl_2 \cdot 2H_2O$ concentrations. The concentration of melamine is A) 0.1 mol/L and B) 0.2 mol/L.

Different molar ratios and concentrations of melamine in DMSO and $CuCl_2 \cdot 2H_2O$ in four different media, DMSO, water + DMSO (1:1 volume ratio), DMF + DMSO (1:1 volume ratio) and Methanol + DMSO (1:1 volume ratio) were tested for crystal growth at room temperature.

As observed in Fig. S1-1, crystals are formed only in (1:1) methanol and DMSO media. This could be due to the hydroxyl group (-OH-) of methanol interacting with the metal ions to form a metal-organic structure Chen et al. [2006], or due to a low solubility of melamine in the solution necessary to form high quality crystals Robin et al. [2006]. In fact, as observed experimentally, methanol does not dissolve properly melamine. Alternatively, it can be attributed to high polarity of DMSO, DMF and water as compared to alcohols, which destabilizes the coordination bonds Kettle [2013].

Figure S1-2 shows that there are not significant differences in the crystal morphology and

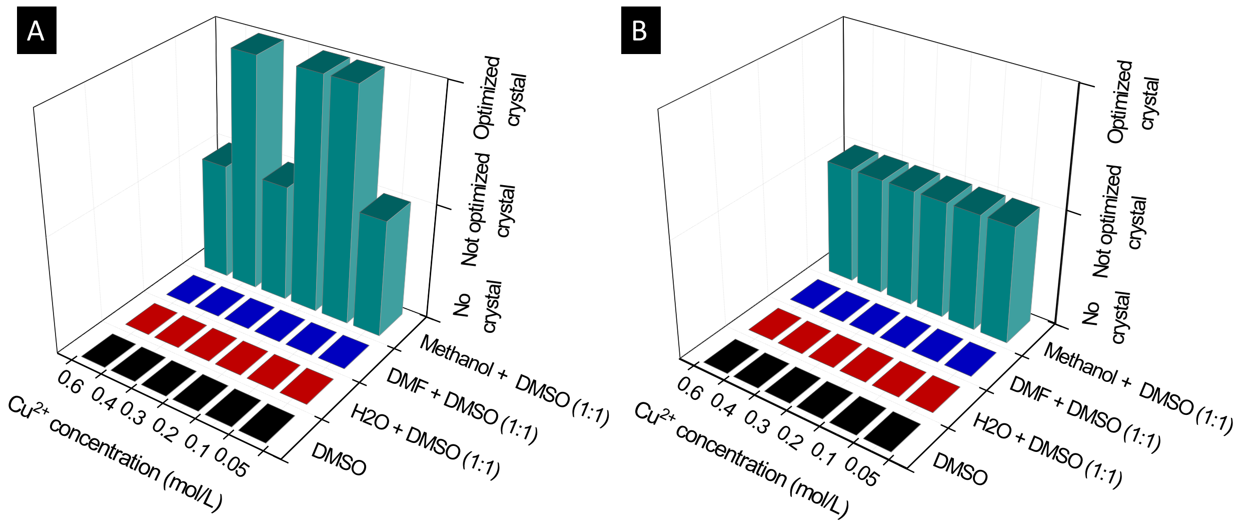

Figure S1-1: Crystal growth at room temperature with different solvents and $CuCl_2$ concentrations. The concentration of melamine is A) 0.1 mol/L and B) 0.2 mol/L.

size between the samples synthesized at room temperature and 50°C. No crystals are formed at 70°C.

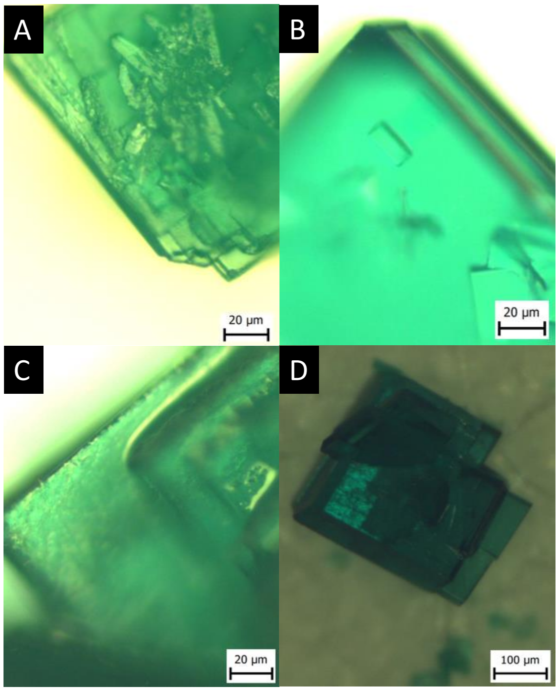

Figure S1-2: Optical micrographs of A) Cu1M1 at 50°C, B) Cu2M1 at 50°C, C) Cu1M1 at room temperature and D) Cu2M1 at room temperature. Cu1M1 stands for 0.1 mol/L of $CuCl_2{\cdot}2H_2O$ in metrhanol and 0.1 mol/L of melamine in DMSO mixed in 1:1 volume ratio. Cu2M1 stands for 0.2 mol/L $CuCl_2{\cdot}2H_2O$ in methanol and 0.1 mol/L of melamine in DMSO mixed in 1:1 volume ratio.

Stability

The stability of the copper-melamine complex and the copper coordination polymer in air and various solvents are showed in Fig. S1-3. The solvents tested are DMSO, DMF, methanol, deionized water, air, acidic media (crystal media but pH 4, reached by adding acetic acid) and basic media (crystal media but al pH 9, reached adding ammonia solution (NH3, 4 mol/L, in methanol).

Chemical stability

To test its chemical resistance to different solvents, the crystal filtered out and rinsed with DMSO was subjected to a 3 mL pure solvent solution in a closed glass vial of 5 mL volume. After a day, the crystal was observed by an optical microscope and compared with a similar one which was left in its forming solution.

pH stability

To test the stability in acid and basic media, acetic acid anhydrous and a solution of ammonium hydroxide in methanol were added to the original solution where the crystals were formed. pH 9 and 4 were reached for basic and acidic cases, respectively. The pH was measured using a pH tester HI98100 Checker Plus of Hanna Instruments.

Air stability

As for their stability in air, the crystals were separated from the supernatant by vacuum filtration. They are washed with the respective suitable solvent and also, they are left on a glass slide for one day. Then, they were observed by an optical microscope and compared with freshly synthesized crystals.

Copper-melamine complex Cu2M1

The copper-melamine complex stands the longest time in DMSO, relatively longer in methanol and air, much shorter in DMF and the shortest in water. The instability in water is common

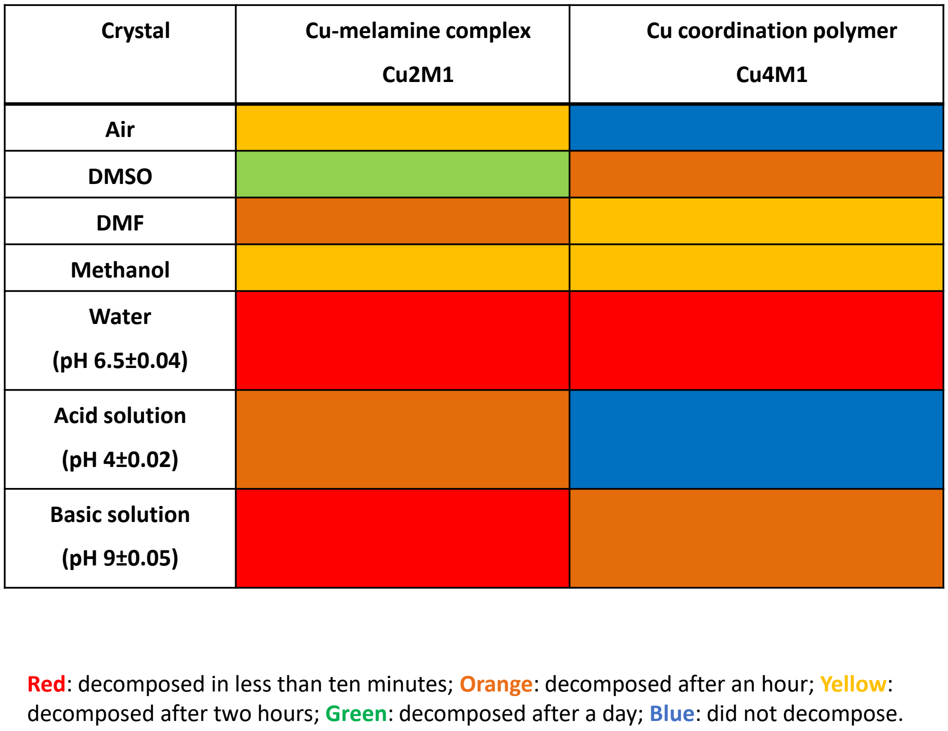

Figure S1-3: The stability of the cupper-melamine complex and the copper coordination polymer in air and various solvents.

in coordination complexes, due the labile nature of coordination bonds which destroy the structure by hydrolysis of the network (Cychosz and Matzger, 2010). The coordination of Cu (II) to N of the triazine ring is weak and susceptible to be attacked by polar solvents. Also, organic solvents do not involve a stable media for these crystals neither. This behavior is characteristic of coordination materials, since polar solvents break the weak bonds that forms their crystalline structures (Kettle, 2013). It is instable in basic systems as the decomposition is caused by the replacement of linkers by OH- that competitively binds to the metal ions. In acid solutions, their poor stability is related to a higher concentration of H+, which exchange with Cu (II) ions in the crystalline structure (Yuan et al., 2018). These properties are associated with ligands, being coordination complexes.

Copper coordination polymer Cu4M1

As shown in Fig. S1-3, the copper coordination polymer is generally more stable than the copper-melamine complex, owing to the strong Cu-Cl. It shows the highest stability in air and acidic conditions.

Crystallization control

Control of the crystallisation of both Cu2M1 and Cu4M1 in the same media was demonstrated as follows. Cu4M was formed without melamine by mixing 2 mL of Cu (II) 0.4 mol/L in methanol with a 2mL mixture of DMSO and acetic acid with a pH of 9.64$\pm$0.02. Then, 2 mL of a DMSO solution of melamine 0.1 mol/L was added in the solution to reach a copper-to-melamine concentration ratio of 2:1. As observed in Fig.S1-4, the dark green Cu2M1 crystals grow on the surface of the light-green Cu4M crystals as long as 6 hours. The Cu2M1 crystals grow as long as 6 hours reaching a maximum size around 800 μm, which is more than twice as large as 350 μm for Cu2M1 crystals grown with melamine. Also, the Cu2M1 crystals on the surface of Cu4M1 is thicker and less edgy as compared with those grown alone (See Fig. 2 in the manuscript). This phenomenon can be related with the crystallization mechanism. According to Ostwald ripening Ostwald [1900], the crystallization is governed by four steps: dissolution, ripening, regrowth and relaxation. A higher presence of copper ions on the surface of Cu4M1 boosts the regrowth and ripening steps, enlarging the size of the Cu2M1 crystals.

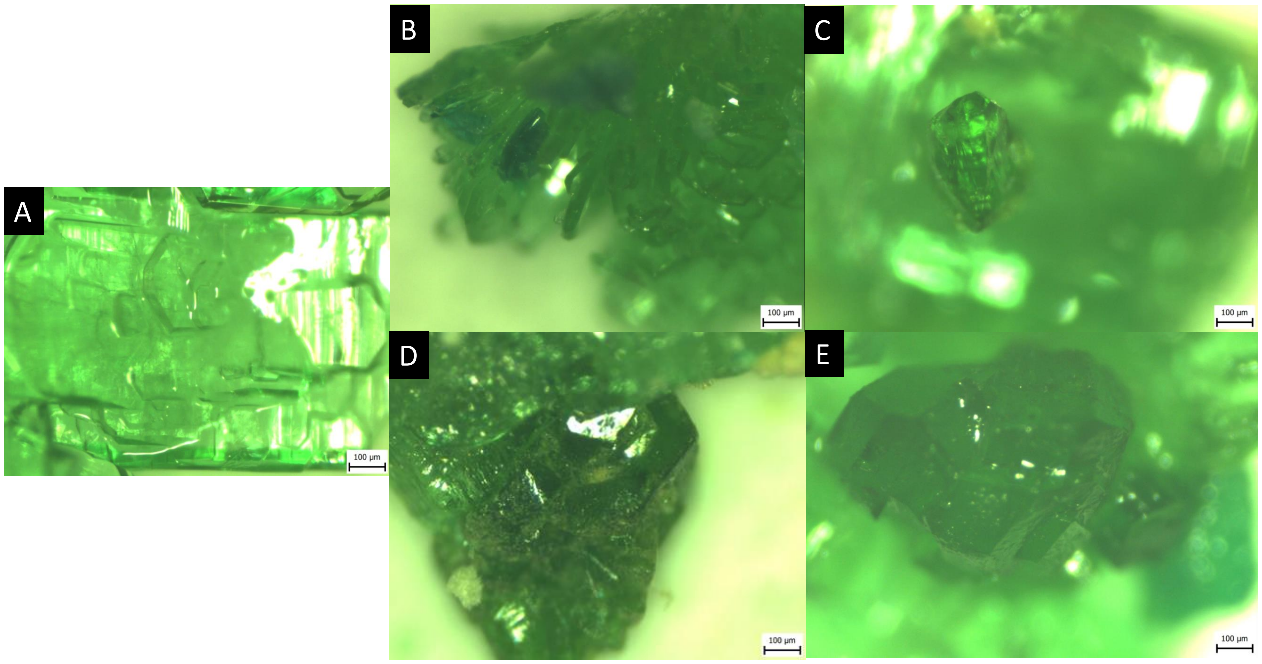

Figure S1-4: Micrographs demonstrating crystallisation of Cu2M1 on the surface of Cu4M1 over time. A) Before adding melamine, B) 1 hour after adding melamine, C) 3 hours after adding melamine, D) 6 hours after adding melamine and E) 24 hours after adding melamine.

Magnetism

Magnetic susceptibility

The temperature dependent magnetizatic susceptibility of the Cu4M1 can be fitted by the following equation derived for a S=1/2 Ising chain Fisher [1964]:

$$\chi_{Cu} = \frac{g_S^2\mu_B^2}{12k_BT}\frac{1+u(K)}{1-u(K)}(1-\zeta) + \frac{g_S^2\mu_B^2}{4k_BT}\zeta, \tag{1}$$

where $u(K) = tanh(K)$ and $K = J/2k_BT$, and ζ corresponds to a concentration of 1/2 paramagnetic impurities or the inverse-temperature term that arises from staggered spins Feyerherm et al. [2000]. As shown in Fig. S1-5, the fitted curve reproduces the peak, but not as good as Eq. 1 in the manuscript for a S=1/2 Heisenberg chain. The least square fit leads to g= 2.201±0.005, J= -0.997±0.007 meV, ζ=0.067±0.001.

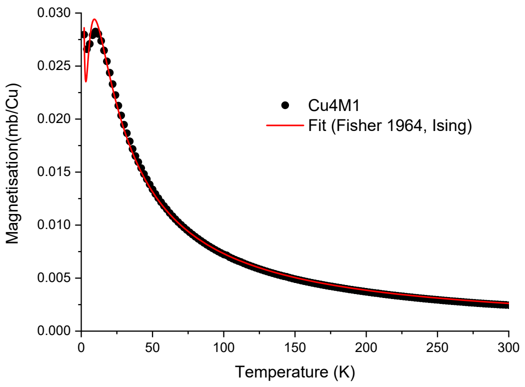

Figure S1-5: Magnetism versus field for dinuclear Cu-melamine compex and Cu coordination polymer

As shown in Fig. S1-6, the best fit can obtained with the well-known rational equation Estes et al. [1978]:

$$\chi_{Cu} = \frac{g_S^2\mu_B^2}{k_BT}\frac{0.25+0.14995x+0.30094x^2}{1+1.9862x+0.68854x^2+6.0626x^3}(1-\zeta) + \frac{g_S^2\mu_B^2}{4k_BT}\zeta. \tag{2}$$

The least square fit leads to g= 2.189±0.002, J= -0.745±0.002 meV, ζ=0.0197±0.0003.

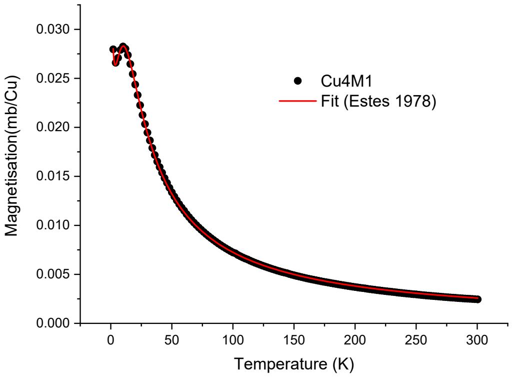

Figure S1-6: Magnetism versus field for dinuclear Cu-melamine compex and Cu coordination polymer

Magnetization isotherms

Figure S1-7 shows the magnetization isotherms for dinuclear Cu-melamine compex Cu2M1 and Cu coordination polymer Cu4M1.

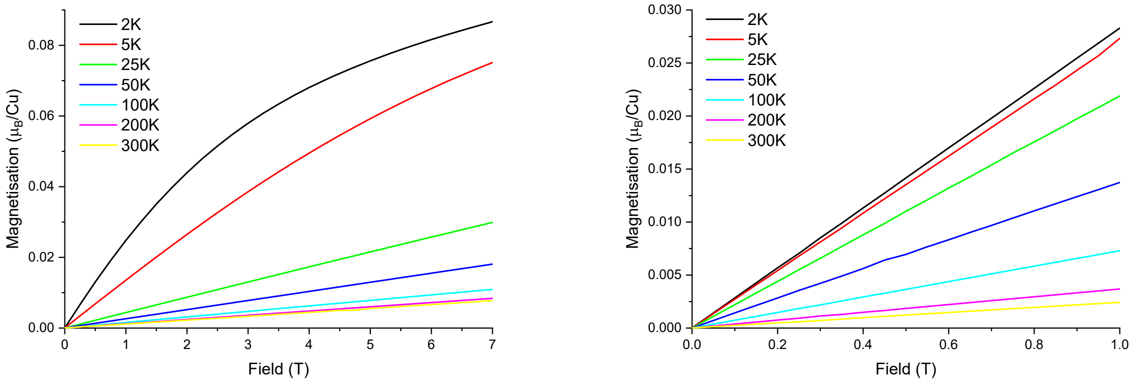

Figure S1-7: Magnetism versus field for dinuclear Cu-melamine compex Cu2M1 (left) and Cu coordination polymer Cu4M1 (right)

The magnetisation of Cu2M1 per Cu saturates well below μ_B= $g_S\sqrt{S(S+1)}\mu_B \simeq 1.73\mu_B$, the effective magnetic moment for a paramagnetic S=1/2 spin. This is due to the impurity (15% of Cu) dominating the net magnetization in the measured temperature range 2–300 K. For both samples, the magnetization isotherm is linear up to 1T so that the Curie or

Curie-Weiss term used in the equations of the magnetic susceptibility is justified.

References

C. Chen, C. W. Yeh, and J. D. Chen. Syntheses, structures and thermal properties of two new copper(II) melamine complexes. *Polyhedron*, 25(6):1307–1312, 2006. ISSN 02775387. doi:10.1016/j.poly.2005.09.020.

W. E. Estes, D. P. Gavel, W. E. Hatfield, and D. J. Hodgson. Magnetic and structural characterization of dibromo- and dichlorobis(thiazole) copper(ii). *Inorg. Chem.*, 17(6): 1415–1421, June 1978. ISSN 0020-1669. doi:10.1021/ic50184a005. URL https://doi.org/10.1021/ic50184a005.

R. Feyerherm, S. Abens, D. Günther, T. Ishida, M. Meißner, M. Meschke, T. Nogami, and M. Steiner. Magnetic-field induced gap and staggered susceptibility in the S= 1/2 chain [PM·cu(NO3)2·(h2o)2]n(PM = pyrimidine). *Journal of Physics: Condensed Matter*, 12 (39):8495–8509, sep 2000. doi:10.1088/0953-8984/12/39/312. URL https://doi.org/10.1088/0953-8984/12/39/312.

M. E. Fisher. Magnetism in one-dimensional systems—the heisenberg model for infinite spin. *American Journal of Physics*, 32(5):343–346, 1964. doi:10.1119/1.1970340. URL https://doi.org/10.1119/1.1970340.

S. F. A. Kettle. *Physical Inorganic Chemistry: A Coordination Chemistry Approach*, volume 11. 2013. ISBN 3662251914. URL https://books.google.com/books?id=14nnCAAAQBAJ{&}pgis=1.

W. Ostwald. *Über die vermeintliche Isomerie des roten und gelben Quecksilberoxyds und die Oberflächenspannung fester Körper*, volume 34U. 1900. doi:10.1515/zpch-1900-3431.

A. Y. Robin, J. L. Sagué, and K. M. Fromm. On the coordination behaviour of NO3- in coordination compounds with Ag+: Part 1. Solubility effect on the formation of coordination polymer networks between AgNO3 and L (L = ethanediyl bis(isonicotinate) as a function of solvent. *CrystEngComm*, 8(5):403–416, 2006. ISSN 14668033. doi:10.1039/b517191a.
