# Supplementary material for: Crystal engineering with copper and melamine
Source: RSC Adv. 2021 Jul 7;11(39):23943–7. doi: 10.1039/d1ra02903g (PMC8262272; doi:10.1039/d1ra02903g)
Supplement: RA-011-D1RA02903G-s002 [file RA-011-D1RA02903G-s002.pdf]

Electronic Supplementary Material (ESI) for RSC Advances.
This journal is © The Royal Society of Chemistry 2021

Electronic Supplementary Information 2

Crystal engineering with copper and melamine

Ignacio Bernabé Vírseda,[a] Shiraz Ahmed Siddiqui,[b] Alexander Prado-Roller,[c] Michael Eisterer,[d] Hidetsugu Shiozawa*[a,b]

[a] J. Heyrovsky Institute of Physical Chemistry, Czech Academy of Sciences, Dolejskova 3, 182 23 Prague 8, Czech Republic. Tel: +420-26605-3755; E-mail: hide.shiozawa@jh-inst.cas.cz

[b] Faculty of Physics, University of Vienna, Boltzmanngasse 5, 1090 Vienna, Austria. Fax: +43-1-4277-9726; Tel: +43-1-4277-72601; E-mail: hidetsugu.shiozawa@univie.ac.at

[c]Institute of Inorganic Chemistry, Faculty of Chemistry, University of Vienna, Währinger Straße 42, Austria

[d]Atominstitut, TU Wien, Stadionallee 2, 1020 Vienna, Austria

X-ray Analysis

The X-ray intensity data were measured on Bruker D8 Venture diffractometer equipped with multilayer monochromator, Mo K/α INCOATEC micro focus sealed tube and Oxford cooling system. The structures were solved by *Direct Methods and Intrinsic Phasing*. Non-hydrogen atoms were refined with *anisotropic displacement parameters*. Hydrogen atoms were inserted at calculated positions and refined with riding model. The following software was used: *Bruker SAINT software package*[i] using a narrow-frame algorithm for frame integration, *SADABS*[ii] for absorption correction, *OLEX2*[iii] for structure solution, refinement, molecular diagrams and graphical user-interface, *Shelxle*[iv] for refinement and graphical user-interface *SHELXS-2015*[v] for structure solution, *SHELXL-2015*[vi] for refinement, *Platon*[vii] for symmetry check. Experimental data and CCDC-Codes Experimental data (Available online: http://www.ccdc.cam.ac.uk/conts/retrieving.html) can be found in Table 1. Crystal data, data collection parameters, and structure refinement details are given in Tables 2 to 5. Table 6 illuminates the trigonal bipyramid to square pyramid range of the compounds and selected data. Asymmetric Unit and packing views visualized in Figures 1 to 5.

Table 1 Experimental parameter and CCDC-Code.

Sample	Machine	Source	Temp.	Detector Distance	Time/ Frame	#Frames	Frame width	CCDC
	Bruker		[K]	[mm]	[s]		[°]	
**Cu4M1**	D8	Mo	100	50	1	835	0.360	2061869
**Cu2M1**	D8	Mo	100	40	5	1683	0.360	2061868

Cu4M1

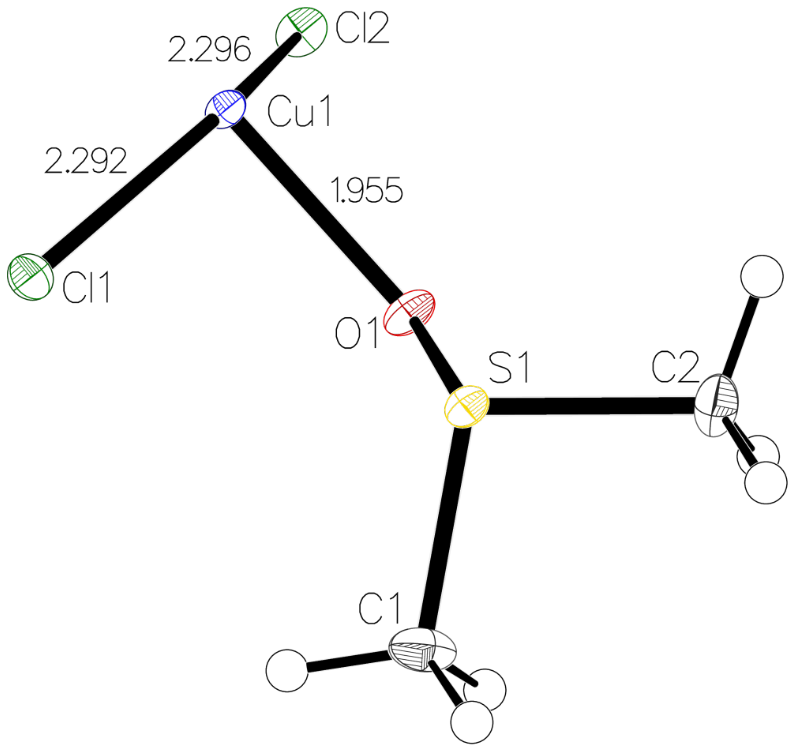

Figure 1 Asymmetric Unit **[Cu4M1]** drawn with 50% displacement ellipsoid. The bond precision for C-C single bonds is 0.0014Å. More detailed information about the coordination geometry of the Copper can be found in table **[Table6]**.

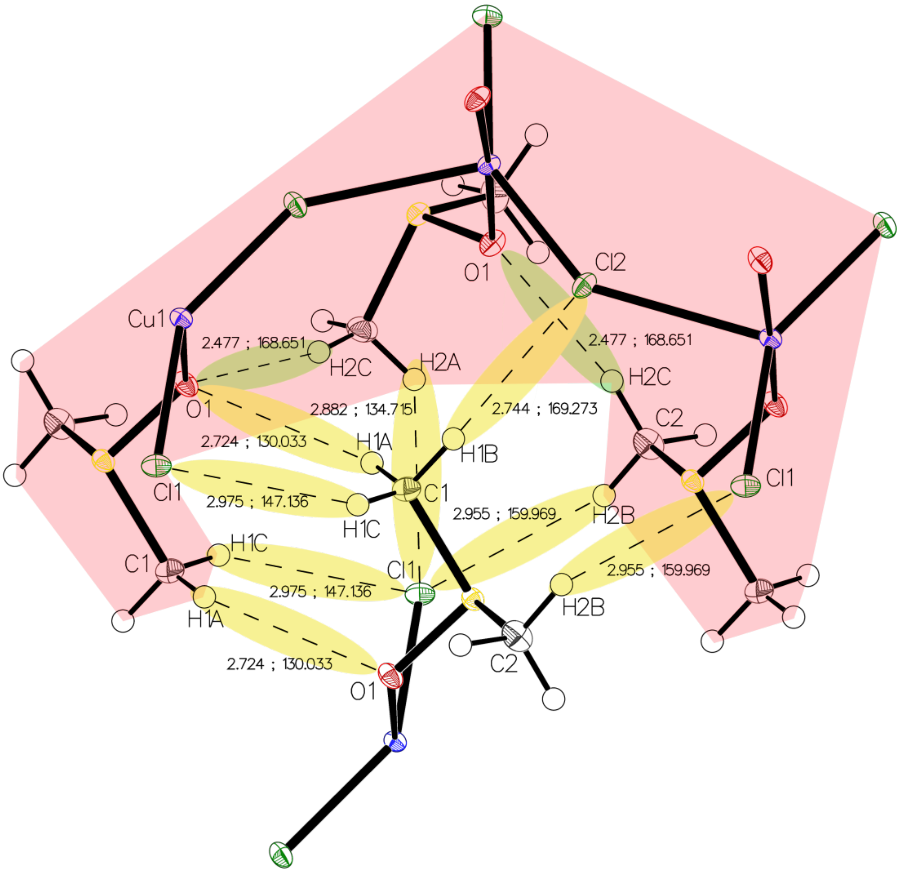

Figure 2 In this packing, it stands out that the connections with chlorine on the copper form one dimensional linear chain and result in a (red marked) "cap". In the area marked in red, two other intramolecular bonds have also been detected (green shaded). The cap encloses a neighbouring strain and it is characterised by several intermolecular interactions (yellow shaded). In addition to the bond lengths [Å] of the inter- and intramolecular bonds, the bond angles [°] are also given.

Table 2 Sample and crystal data. **[Cu4M1]**

Radiation [Å]	MoKα (λ = 0.71073)	Z	4	Measurement method	\f and \w scans
Crystal habit	clear blue block	a [Å]	7.9376(3)		
Crystal size [mm^3]	0.2 × 0.2 × 0.15	b [Å]	11.4925(4)	Abs. correction type	multiscan
Empirical formula	C4H12Cl2CuO2S2	c [Å]	11.3612(4)	Abs. correction Tmin	0.2120
Formula weight [g/mol]	290.70	α [°]	90	Abs. correction Tmax	0.2650
Temperature [K]	100.0	β [°]	90	Density (calculated) [g/cm3]	1.863
Crystal system	Orthorhombic	γ [°]	90	Absorption coefficient [mm^{-1}]	2.979
Space group	Pnma	Volume [$Å^3$]	1036.40(6)	F (000) [e^-]	588.0

Table 3 Data collection and structure refinement. **[Cu4M1]**

2Θ range for data collection [°]	5.042 to 60.106	Index ranges		Goodness-of-fit on F^2	1.093
Reflections collected	6336	h	-11 ≤ h ≤ 9	Diff. peak and hole [$e^-Å^{-3}$]	0.43/-0.57
Data / restraints / parameters	1587/0/57	k	-14 ≤ k ≤ 16		
Refinement method	Direct Methods	l	-16 ≤ l ≤ 13	Function minimized	$\Sigma w (F_o^2 - F_c^2)^2$
		all data	R1 = 0.0223, wR2 = 0.0495	Weighting scheme	where
		I>2σ(I)	R1 = 0.0199, wR2 = 0.0486	$w=1/[\sigma^2(Fo^2) + (0.0212P)^2 + 0.3233P]$	$P=(F_o^2+2F_c^2)/3$

Cu2M1

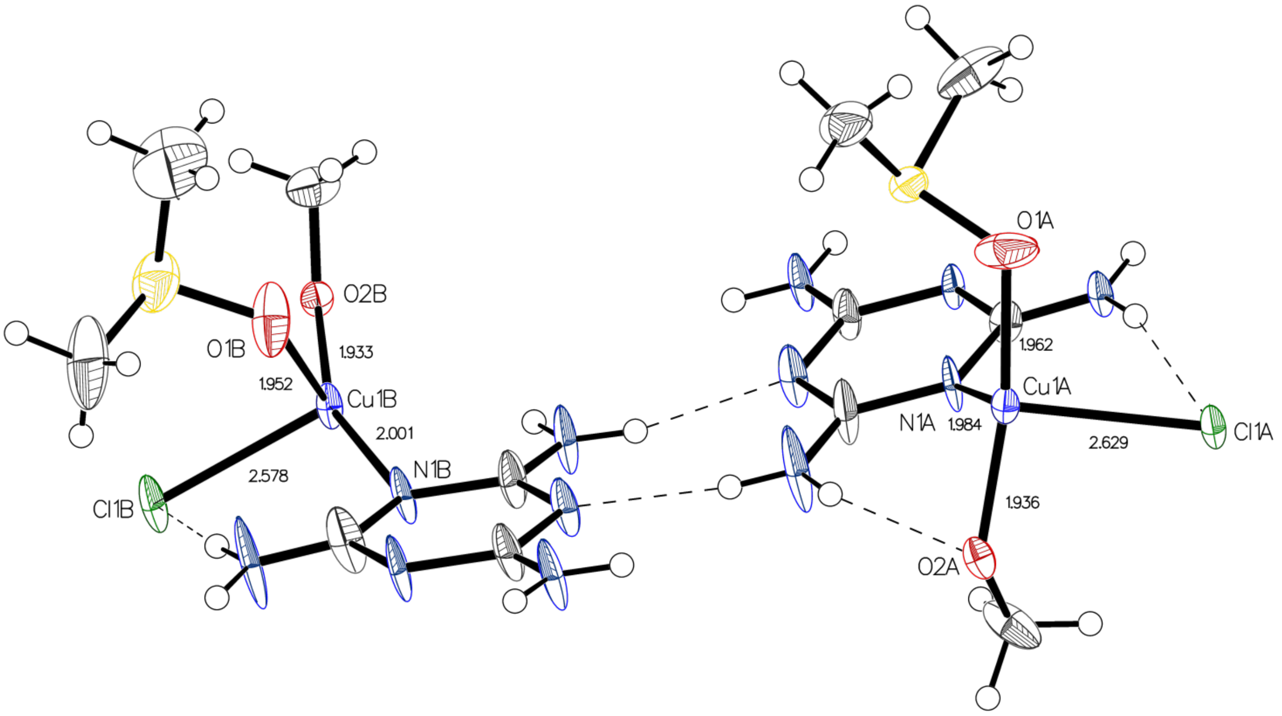

Figure 3 Crystal structure **[Cu2M1]** drawn with 50% displacement ellipsoid. The bond precision for C-C single bonds is 0.0070Å. Main residue disorder is 12%. Squeeze was used because of high degree of disorder in the co-crystalized solvent. Here, the hydrogen bonds between two independent molecules are visualized by stippled lines at the nitrogen's. Also the intramolecular interactions from Nitrogen to Chloride and Nitrogen to Oxygen are visualized in the same way. More detailed information about the coordination geometry of the Copper can be found in table **[Table6]**. The platon report picks out A and B Alerts of PLAT213. This is related to a fact that is visible in the figure. The big ratio of the ADP's between the different axes, especially the value of the perpendicular elongation to the aromatic plane of the coordinated melamine is unusual high. The good overall measurement quality of the crystal excludes artificial effects. It seems that the strong perpendicular elongation to the aromatic plane is real characteristic of the structure and the A and B Alerts must be interpreted in that way. The interpretation of continuous disorder is based on the experience during the refinement that two or more different separated positions of the melamine ligand could not be realised in a stable way. A similar system with melamine ligands is already reported on the CCDC 134810. The behaviour of the ADP's on the melamine was ordinary and not asymmetric like pictured above.

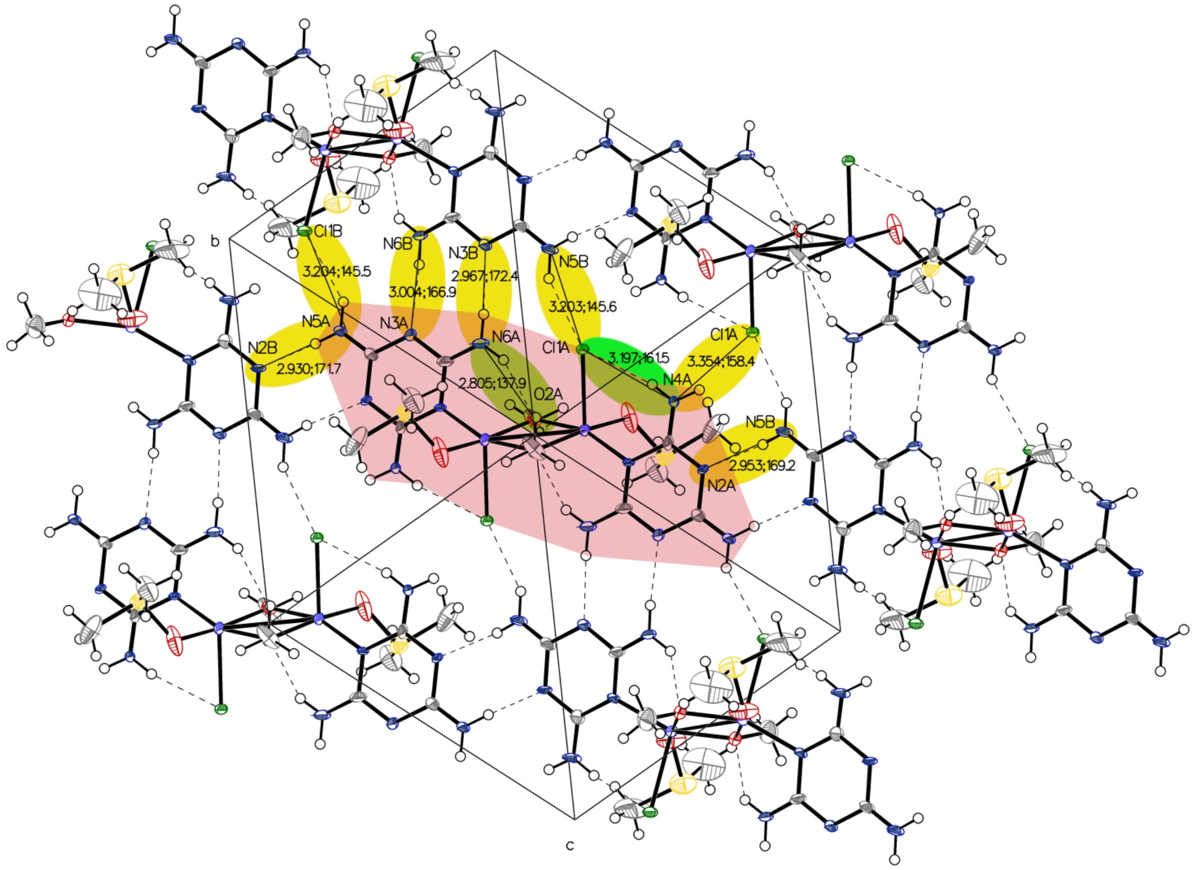

Figure 4 Packing view along 1 1 1 shows that every dimer (red) is surrounded by six neighbouring dimers in the plane. The plane is characterised by intermolecular interactions (yellow). Also two intramolecular interactions visualized (green).

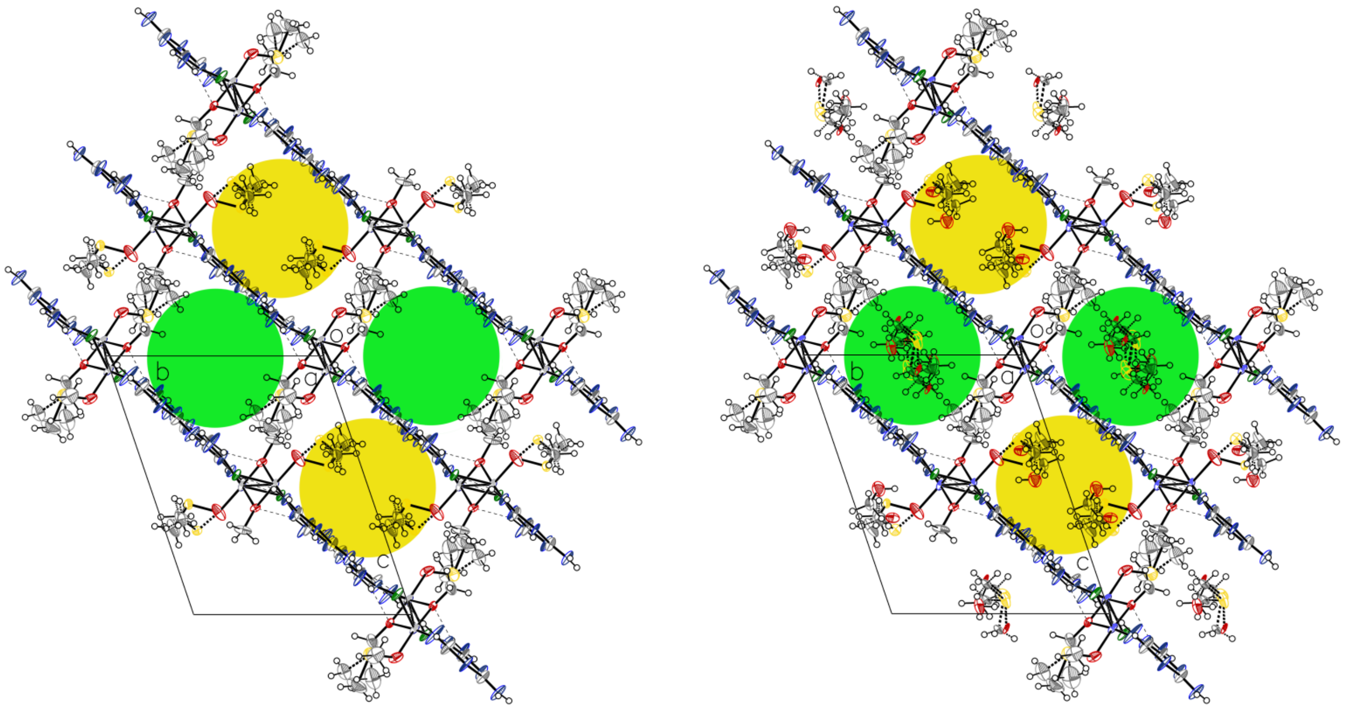

Figure 5 Packing along 1 0 0 makes solvent accessible voids visible. At the left hand the squeezed model without co-crystallised solvents and on the right side a solvent filled model is pictured. Two different types of similar sized voids could be detected. The green shaded void is along the visible radius almost not sterically influenced. Because of this two different types of solvents (DMSO and MeOH) used during the synthesis co-crystallised in this void in a disorded way. The second type of void (yellow) is influenced by the coordinated DMSO and limits the available space. Only MeOH can be modelled. The final model on the CCDC did not contain any free solvent. The strong necessary use of constrains and restrains to fix solvent atom positions made the use of squeeze more serious.

Table 4 Sample and crystal data. **[Cu2M1]**

Radiation [Å]	MoKα (λ = 0.71073)	Z	4	Measurement method	\f and \w scans
Crystal habit	clear green block	a [Å]	10.4677(14)		
Crystal size [mm^3]	0.05 × 0.04 × 0.04	b [Å]	11.580(2)	Abs. correction type	multiscan
Empirical formula	C6H15ClCuN6O2S	c [Å]	14.375(3)	Abs. correction Tmin	0.2319
Formula weight [g/mol]	334.29	α [°]	106.116(8)	Abs. correction Tmax	0.2650
Temperature [K]	100.0	β [°]	99.250(8)	Density (calculated) [g/cm3]	1.395
Crystal system	Triclinic	γ [°]	102.092(8)	Absorption coefficient [mm^{-1}]	1.671
Space group	P-1	Volume [$Å^3$]	1591.8(5)	F (000) [e^-]	684.0

Table 5 Data collection and structure refinement. **[Cu2M1]**

2Θ range for data collection [°]	4.026 to 50.684	Index ranges		Goodness-of-fit on F^2	1.103
Reflections collected	27969	h	$-12 \le h \le 12$	Diff. peak and hole [$e^- Å^{-3}$]	1.36/-1.24
Data / restraints / parameters	5796/259/329	k	$-13 \le k \le 13$		
Refinement method	Intrinsic Phasing	l	$-17 \le l \le 17$	Function minimized	$\Sigma\ w\ (F_o^2 - F_c^2)^2$
		all data	R1 = 0.0616, wR2 = 0.1349	Weighting scheme	where
		I>2σ(I)	R1 = 0.0499, wR2 = 0.1295	$w=1/[\sigma^2(Fo^2) + (0.0644P)^2 + 3.8284P]$	$P=(F_o^2+2F_c^2)/3$

Table 6 Data mining at the CCDC for similar structure types of Cu2M1, top table, & Cu4M1, bottom table, of trigonal bipyramid's or square pyramid's with similar ligand sphere can only show a possible range of metal-metal interaction and the influence on the connecting oxygen to the manifestation of Tau, the used parameter to determine the ratio between trigonal bipyramid or square pyramid. Final conclusion is that the main geometry seems to be the square pyramid (convergence to 0). A real rule for Cu2M1 similar and Cu4M1 similar compounds cannot be fixed.

CCDC	Tau-value*	Coordination-partner	Interaction metal-metal	Angle Cu-O-Cu
**2061868 (CuA)**	0.12	-O, -O, -O, -N, -Cl	Cu(1A)-Cu(1A) = 3.0122	Cu(1A)-O(2A)-Cu(1A) = 102.73(13)
**2061868 (CuB)**	0.19	-O, -O, -O, -N, -Cl	Cu(1B)-Cu(1B) =3.0050	Cu(1B)-O(2B)-Cu(1B) = 102.14(16)
**1429521**	0.23	-O, -O, -O, -N, -N	Cu-Cu = 2.9962	Cu-O(2)-Cu = 102.27(12)
**740504**	0.13	-O, -O, -O, -N, -Cl	Cu-Cu = 3.0354	Cu-O(2)-Cu = 103.72(8)
**740503**	0.12	-O, -O, -O, -N, -Cl	Cu-Cu = 3.0434	Cu(1)-O(1)-Cu(1) = 103.76(5)
**1935842 (Cu1)**	0.35	-O, -O, -O, -N, -Cl	Cu(1)-Cu(2) = 3.0122	Cu(1)-O(26)-Cu(2) = 99.76(6)
**1935842 (Cu2)**	0.21	-O, -O, -N, -Cl, -Cl	Cu(1)-Cu(2) = 3.0122	Cu(1)-O(15)-Cu(2) = 102.99(6)
**890384 (Cu1)**	0.42	-O, -N, -Cl, -Cl, -Cl	Cu(1)-Cu(2)= 2.9356	Cu(1)-O(1)-Cu(2) = 97.44 (7)
**890384 (Cu2)**	0.26	-O, -N, -Cl, -Cl, -Cl	Cu(1)-Cu(2) = 2.9357	Cu(1)-O(1)-Cu(2) = 97.44 (7)
**2061869**	0.45	-O, -O, -Cl, -Cl, -Cl		
**1576053**	0.18	-O, -N, -Cl, -Cl, -Cl		
**1576054**	0.35	-O, -N, -Cl, -Cl, -Cl		
**1576055**	0.05	-O, -N, -Cl, -Cl, -Cl		
**602083 (Cu3)**	0.21	-O, -O, -N, -Br, -Br		
**1935841**	0.21	-O, -N, -N, -N, -Cl		
**1429519**	0.55	-O, -O, -N, -N, -Cl		
**602083 (Cu1)**	0.1	-O, -O, -O, -N, -Br		
**602083 (Cu2)**	0.09	-O, -O, -O, -N, -Br		

Tau-value*= Tau-Descriptor for 5-Coordination[viii]

[i] Bruker SAINT v8.38B Copyright © 2005-2019 Bruker AXS

[ii] Sheldrick, G. M. (1996). *SADABS.* University of Göttingen, Germany.

[iii] Dolomanov, O.V., Bourhis, L.J., Gildea, R.J, Howard, J.A.K. & Puschmann, H. , OLEX2, (2009), J. Appl. Cryst. 42, 339-341

[iv] C. B. Huebschle, G. M. Sheldrick and B. Dittrich, ShelXle: a Qt graphical user interface for SHELXL, J. Appl. Cryst., 44, (2011) 1281-1284

[v] Sheldrick, G. M. (2015). *SHELXS v 2016/4* University of Göttingen, Germany.

[vi] Sheldrick, G. M. (2015). *SHELXL v 2016/4* University of Göttingen, Germany.

[vii] A. L. Spek, Acta Cryst. 2009, D65, 148-155

[viii] A.W.Addison,T.N.Rao,J.Reedijk,J.van Rijn,G.C.Verschoor, J.Chem.Soc.Dalton Trans.(1984),1349-1356
